# Primary hypertrophic osteoarthropathy: genetics, clinical features and management

**DOI:** 10.3389/fendo.2023.1235040

**Published:** 2023-08-29

**Authors:** Qi Lu, Yang Xu, Zeng Zhang, Shanshan Li, Zhenlin Zhang

**Affiliations:** ^1^ Shanghai Clinical Research Center of Bone Disease, Department of Osteoporosis and Bone Diseases, Shanghai Jiao Tong University Affiliated Sixth People’s Hospital, Shanghai, China; ^2^ Department of Orthopedic Surgery, Shanghai Jiao Tong University Affiliated Sixth People’s Hospital, Shanghai, China

**Keywords:** primary hypertrophic osteoarthropathy, prostaglandin E2, NSAIDs, SLCO2A1 gene, HPGD gene

## Abstract

Primary hypertrophic osteoarthropathy (PHO) is a genetic disorder mainly characterized by clubbing fingers, pachydermia and periostosis. Mutations in the *HPGD* or *SLCO2A1* gene lead to impaired prostaglandin E2 (PGE2) degradation, thus elevating PGE2 levels. The identification of the causative genes has provided a better understanding of the underlying mechanisms. PHO can be divided into three subtypes according to its pathogenic gene and inheritance patterns. The onset age, sex ratio and clinical features differ among subtypes. The synthesis and signaling pathways of PGE2 are outlined in this review. Cyclooxygenase-2 (COX-2) is the key enzyme that acts as the rate-limiting step for prostaglandin production, thus COX-2 inhibitors have been used to treat this disease. Although this treatment showed effective results, it has side effects that restrain its use. Here, we reviewed the genetics, clinical features, differential diagnosis and current treatment options of PHO according to our many years of clinical research on the disease. We also discussed probable treatment that may be an option in the future.

## 1 Introduction

Primary hypertrophic osteoarthropathy (PHO) is an inherited disorder of skeletal and skin abnormalities. The history of the disease dates back to 1868, when Friedreich first described “hyperostosis of the entire skeleton” in two brothers (1). Less than a century later, Albert Touraine, Gabriel Solente, and Laurent Golé distinguished primary hypertrophic osteoarthropathy from secondary hypertrophic osteoarthropathy (SHO), which usually associated with an underlying cause such as pulmonary or cardiac diseases (2). Hence, the term Touraine-Solente-Gole syndrome is also used for this disease. There are currently two accepted classifications of the disease. The first is based on clinical manifestation, and three clinical subtypes were proposed: (i) the complete form, presenting the full-blown phenotype; (ii) the incomplete form, with isolated bone involvement and limited skin changes; and (iii) the fruste form, with pachydermia and minimal or absent periostosis (3). The other classification occurred after the identification of the two disease causal genes, the *HPGD* gene and *SLCO2A1* gene, which encodes 15-hydroxyprostaglandin dehydrogenase (15-PGDH) and solute carrier organic anion transporter family member 2A1 (OATP2A1, also known as prostaglandin transporter, PGT), respectively. At present, it can be divided into three subtypes based on its inheritance pattern and causal genes: 1) primary hypertrophic osteoarthropathy, autosomal recessive 1 (PHOAR1, MIM 259100); 2) primary hypertrophic osteoarthropathy, autosomal recessive 2 (PHOAR2, MIM 614441); and 3) primary hypertrophic osteoarthropathy, autosomal dominant (PHOAD, MIM 167100). PHOAR1 is caused by *HPGD* gene mutation, while *SLCO2A1* gene is the causal gene of the other two types. This article provides an overview of the genetics and pathophysiology of PHO, as well as diagnosis and treatment based on our long-term study on this disease for one decade.

## 2 Genetics and pathogenesis

As early as the 19th century, PHO was found to have a familial tendency. The inheritance pattern of PHO has long been discussed before the pathogenic gene was discovered. Both autosomal recessive and autosomal dominant patterns with variable penetrance were proposed. Castori et al. reviewed 204 PHO patients reported between 1965 and 2004 and confirmed that 54.4% of them showed autosomal dominant transmission, and the remaining showed an autosomal recessive form (4). In 2008, Uppal et al. identified *HPGD* gene as the causal gene of PHOAR1 (5). Later in 2012, our team found another gene, *SLCO2A1* gene, that can cause PHOAR2 (6). Recently, in 2021, our team reported that *SLCO2A1* monoallelic mutations are the pathogenic cause of PHOAD with marked interfamilial and intrafamilial variability and then further verified that PHOAR2 probands and PHOAD parents coexisted in the same families, which illustrated the allelic nature of PHOAD and PHOAR2 (7). To date, the causal gene of both autosomal recessive and autosomal dominant PHO has been established. The identification of the pathogenic genes for PHO initiated a burst of exciting new information about the mechanism of this disease, which links the pathogenesis of PHO to the prostaglandin metabolic pathway.

### 2.1 *HPGD* and *SLCO2A1* genes

**Figure 1:**
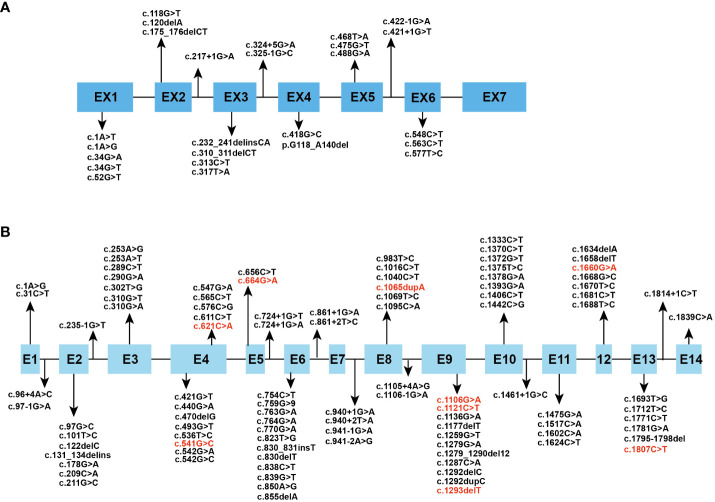
Spectrum of reported **(A)** *HPGD* and **(B)** *SLCO2A1* gene mutation. The mutational sites which can cause PHOAD are in red font.

The human *HPGD* gene is located on chromosome 4q34–q35 and consists of 7 exons (8). It encodes 15-PGDH, the key enzyme responsible for biological inactivation of prostaglandins, which mediates the initial oxidation of the 15-hydroxyl group (9). 15-PGDH is a dimer consisting of two identical subunits with a relative molecular weight of approximately 28975 Da (10). It is a 266-amino acid protein that is ubiquitously expressed in mammalian tissues. Whole-genome integrated transcriptomics and antibody proteomics revealed relatively high levels of *HPGD* gene expression in the bladder, stomach, duodenum, lung, small intestine, placenta, liver, esophagus, colon, and kidney in human tissues (11). The human *SLCO2A1* gene is located on chromosome 3q22.1-q22.2 and consists of 14 exons (12). It encodes OATP2A1, which serves as a prostaglandin transporter. It consists of 643 amino acids and contains 12 transmembrane domains and is expressed in human tissues mainly in the thyroid gland, lung, kidney, seminal vesicle, ductus deferens and placenta (13). Prostaglandin catabolism first requires transport into the cell by PGT. It can also help with the release of newly synthesized prostaglandins by mediating the efflux of prostaglandins in exchange for the import of anions such as lactate (14). We reviewed all the mutation sites of the two genes in PHO patients reported thus far, and the results are summarized in **Figure 1** (15–52).

### 2.2 Prostaglandin E2 metabolism and function

Arachidonic acid (AA) resides in the cell membrane and is released from membrane phospholipids catalyzed by phospholipase A2 (PLA2). Then, cyclooxygenase (COX) oxygenates the AAs at the luminal side of nuclear and endoplasmic reticulum (ER) membranes and forms prostaglandin H2 (PGH2), which is then converted into different prostaglandins by prostaglandin synthases (PGS). There are two types of COX enzymes, COX-1 and COX-2, which are similar in structure and catalytic activity. COX-1 is constitutively expressed in the majority of cells. COX-2 is an inducing enzyme that acts as the rate-limiting step for prostaglandin production. COX-2 gene expression can be induced by multiple inflammatory mediators. In PGE2 production, prostaglandin synthases include cytosolic prostaglandin E synthases (cPGES) and microsomal prostaglandin E synthases 1/2 (mPGES-1/2). mPGES-2 and cPGES are constitutively expressed, while mPGES-1 is mainly coupled with COX-2 to increase the production of PGE2. Both PGE2 synthesis and degradation are accomplished intracellularly. However, to perform its major physiological functions, PGE2 usually needs to be transported out of the cell. In addition to simple diffusion, several proteins have PGE2 transport activity, such as multidrug resistance protein 4 (MRP4) and PGT. PGE2 degradation relies on the enzyme 15-PGDH, which only exists in cells. 15-PGDH can degrade PGE2 to prostaglandin metabolite (PGE-M), which presents greatly reduced biological activities. The reuptake and oxidation of PGE2 in the cell leads to signal termination. A two-step model of PGE2 metabolic clearance was proposed: 1) selective PG uptake across the plasma membrane and then 2) oxidation inside the cell (53). Thus, prostaglandin oxidation requires coexpression of the PGT along with 15-PGDH, and the deficiency of either leads to impaired prostaglandin degradation, which in turn leads to elevated prostaglandin levels. PGE2 has a rapid turnover rate *in vivo*. Thus, a good balance between COX2-regulated PGE2 formation and the degradation of PGE2 driven by PGT and 15-PGDH is key for maintaining the physical function of PGE2.

**Figure 2:**
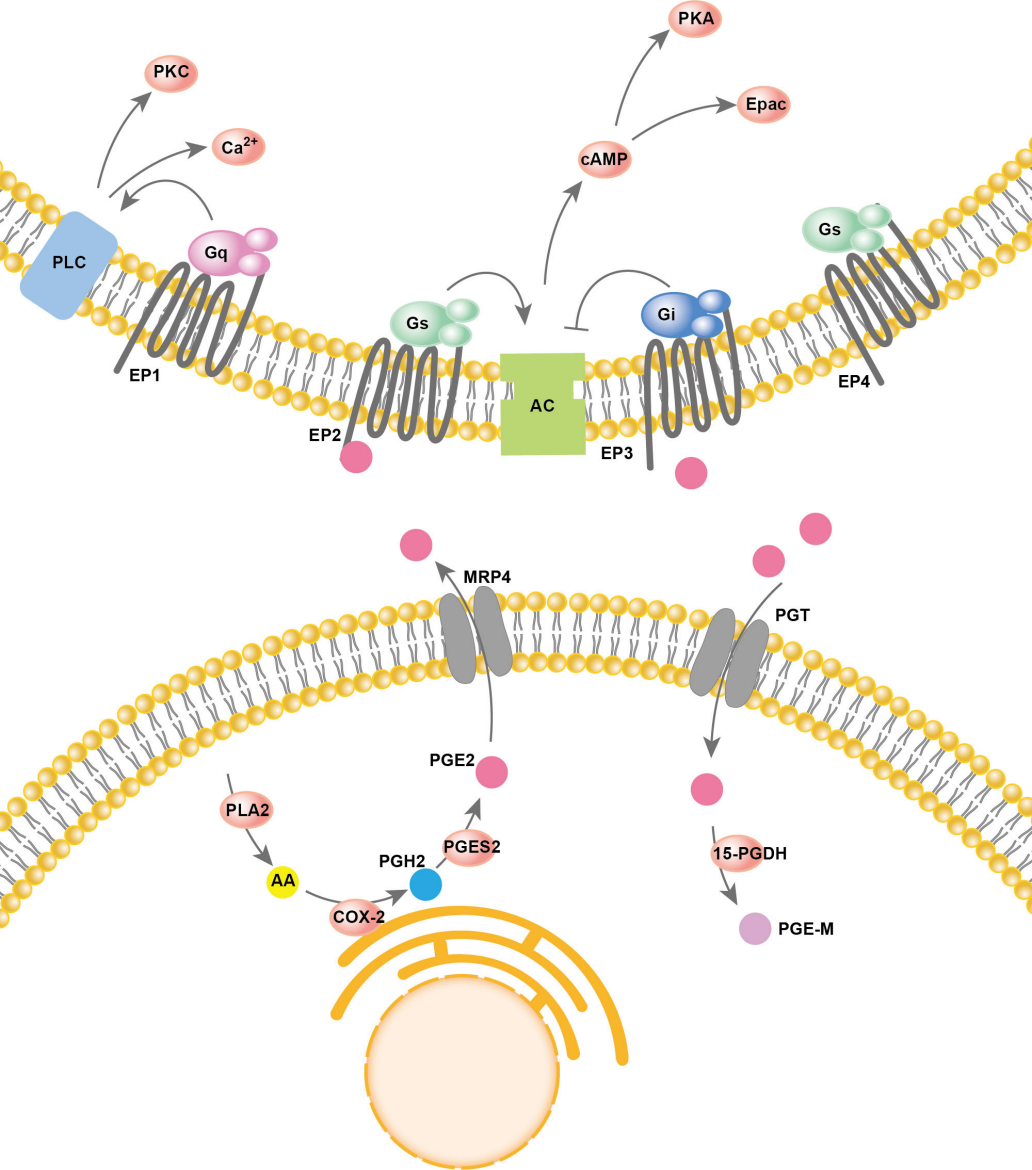
PGE2 synthesis and signaling pathways.

PGE2 is produced by almost all cell types in the human body and plays important roles in various physiological functions, such as activation of endogenous stem cells, angiogenesis, tissue repair, fertility, pain and immune regulation, by activating prostaglandin receptors (54). PGE2 signals through four specific G−protein coupled E−prostanoid (EP) receptors: EP1R, EP2R, EP3R and EP4R. The effect of prostaglandin on different tissues or cell types depends on the expression pattern of each prostaglandin receptor subtype. EP1R acts largely by increasing calcium flux but perhaps also *via* protein kinase C (PKC). Both EP2R and EP4R are coupled to Gαs and stimulate cyclic 3,5-adenosine monophosphate (cAMP) formation. EP3R is coupled to Gαi and acts largely by inhibiting cAMP production (55). The overall process of PGE2 synthesis, degradation, and activation of downstream signaling is summarized in **Figure 2**.

### 2.3 Pathogenesis

Many hypotheses have been proposed to explain the mechanism. Megakaryocytes fragment into platelets in the pulmonary capillary bed. Based on this fact, in 1987, Dickinson and Martin proposed the theory that in diseases where megakaryocytes bypass the pulmonary capillary network, large platelet clumps or chronic platelet overabundance cause large particles to reach the fingertips in axial vascular flow, releasing platelet-derived growth factors (PDGF) (56). PDGF can increase vascular permeability and mediate the inflammatory response, indicating that it may play a role in the genesis of clubbing fingers. Other circulating vasodilators, such as carbon dioxide, ferritin, PGE2, bradykinin, adenine nucleotides (especially ATP), 5-hydroxytryptamine and growth hormone, have also been proposed as candidates (56). Vascular endothelial growth factor (VEGF) can promote angiogenesis and increase vascular wall permeability (57). It also promotes endothelial bone formation and can activate osteoblast function and migration. Plasma levels of VEGF were significantly higher in PHO patients and SHO patients caused by lung cancer, which confirmed the theory that VEGF may play a potential role in hypertrophic osteoarthropathy (HOA) (58, 59). These are the hypotheses suggested to explain the symptoms of PHO before the identification of the disease-causing genes. It is now generally accepted that elevated levels of PGE2 are responsible for the occurrence of PHO. There are connections between PGE2 and these two growth factors: PGE2 can stimulate VEGF expression in endothelial cells (60). PDGF induces the releasing of PGE2 (61). Based on these facts, the role in causing symptoms of elevated PGE2 levels in PHO may be partly through increased VEGF. And the PDGF hypothesis proposed by Dickinson and Martin may work by causing the increasing of PGE2 levels in SHO patients. Further investigation is needed to figure this out.

Histological studies have provided evidence of pathogenesis. Deposition of collagen fibers in soft tissue, interstitial edema, dilated small blood vessels, vascular wall hyperplasia and perivascular lymphocyte infiltration are the pathological findings of digital clubbing (62). Skin biopsy from the thickened forehead showed dermal edema, marked infiltration of mast cells in the dermis, mucin deposition, elastic fibrosis, sebaceous hypertrophy and hyperplasia (16, 63, 64). For joints, minimal synovial cell proliferation but prominent arterial-wall thickening, with intravascular deposition of electron-dense material, were found. Synovial effusions are noninflammatory and show elevated synovial fluid fibrinogen levels (65). Synovial biopsy reveals collagen fiber proliferation creating densified bundles and the formation of a new microvascular network with enlarged endothelial cells. The proliferation of endothelial cells causes notable vascular fibrosis and stenosis (66). Electron-dense deposits in vessel walls suggest that circulating materials have a role in pathogenesis. Many authors have observed increased fibroblast proliferation in bone marrow biopsies associated with diffuse dermal endothelial hyperplasia, partial occlusion of the vascular lumen, pericapillary lymphohistiocytic infiltrate and thickening and packaging of collagen fibers. Affected tissues in PHO patients share similar pathological changes, namely, edema, localized endothelial hyperplasia, and excessive collagen deposition, which may reflect the pathogenesis. Histological studies have shown a lack of inflammatory and autoimmune evidence.

At present, it is generally accepted that prostaglandins, especially PGE2, play important roles in the development of PHO. Mutations in either the *HPGD* gene or *SLCO2A1* gene lead to abnormal protein function, impaired PGE2 degradation and elevated PGE2 levels. PGE2 is associated with cell proliferation and angiogenesis, which are seen in the histological changes in clubbing fingers or skin biopsies in PHO patients. In addition, the relationship between PGE2 and bone has long been discussed. Prostaglandins are known to play a crucial role in maintaining bone homeostasis. COX-2 knockout animals have decreased bone density, and the conditional knockout of COX-2 in osteoblasts shows defective bone microstructure. Both osteoclast and osteoblast function can be promoted by PGE2 (67). These results confirmed that prostaglandins produced by osteoblasts are necessary for maintaining balanced bone turnover. Recently, a novel pathway connecting the skeletal system and neuron system has been discovered (68). It was proposed that PGE2 generated by osteoblasts activates the EP4 receptor on sensory nerves and regulates sympathetic nerves to secrete NE through the CNS. The patients’ characteristic skeletal phenotype of PHO leads us to wonder the specific role of PGE2 in the skeletal system. Elucidation of the underlying mechanism of periostosis in PHO patients induced by high PGE2 levels will help us to propose novel treatments for PHO and other common diseases, such as osteoporosis. The exact effect of prostaglandins on bone and periosteum remains to be elucidated. The unraveling of the underlying mechanism of PHO makes us to wonder if the elevating PGE2 levels may also blamed for the secondary form. PGE2 degrade mainly in the lung. And most of the diseases that can cause HOA have altered lung function. Another evidence that can support this theory is that several SHO patients were treated successfully with COX-2 inhibitors. More work should be done to elucidate the pathogenesis of SHO.

## 3 Clinical features

**Figure 3:**
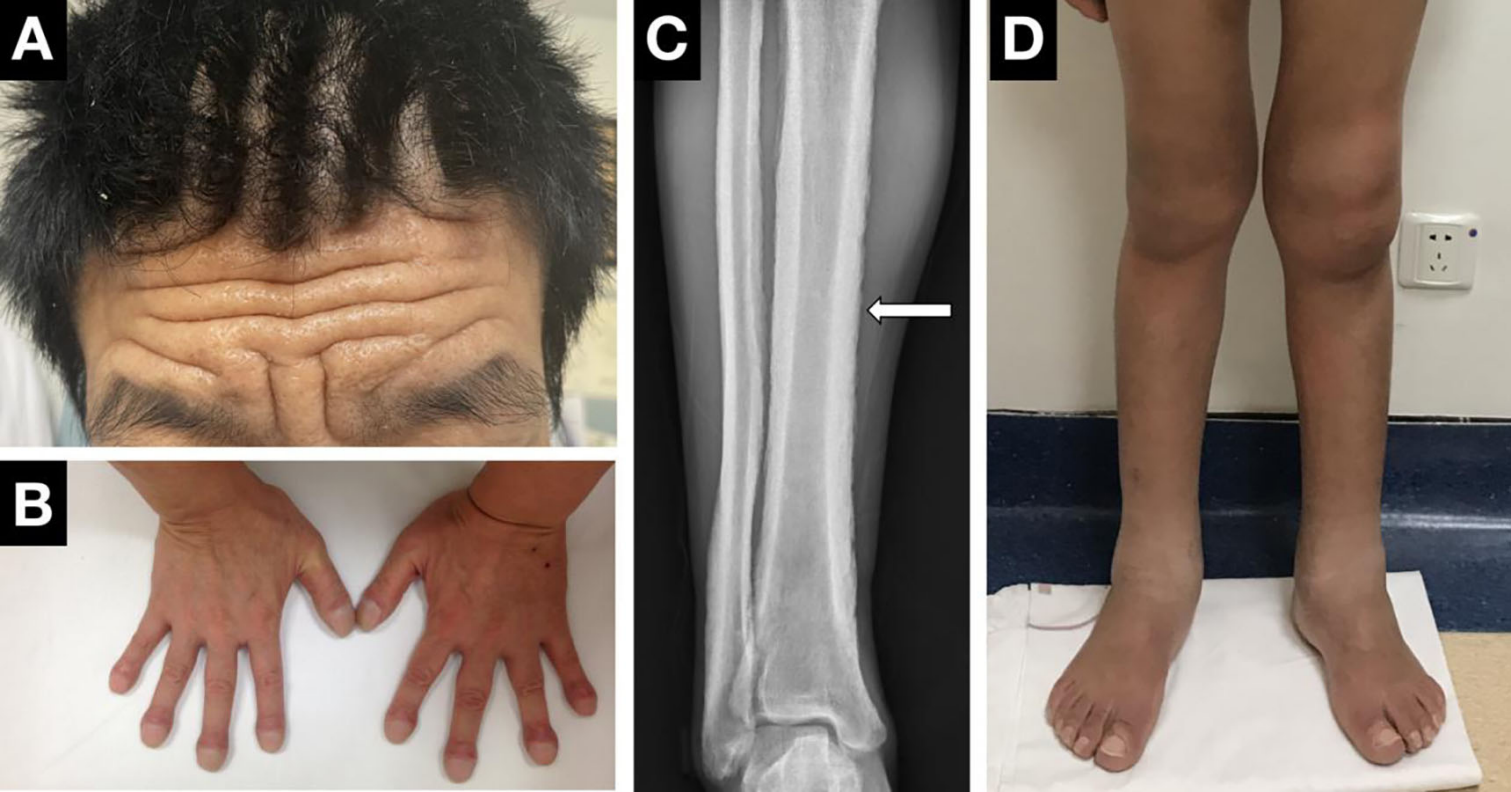
Clinical manifestations of PHO. **(A)** Pachydermia and cutis verticis gyrate. **(B)** Digital clubbing. **(C)** X-ray performance of periostosis. **(D)** Articular manifestations of the knees and ankle.

Digital clubbing, pachydermia and periostosis are considered the most common triad of PHO (**Figure 3**). Clubbing, first described by Hippocrates over 2500 years ago, is characterized by painless enlargement at the distal parts of the extremities, affecting the daily use and appearance of the fingers. It occurs in almost all PHO patients, usually as the initial symptom, and sometimes may be the only manifestation. Clubbing fingers can be diagnosed and assessed by different objective signs, including profile sign, hyponychial angle, and phalangeal depth ratio (69). The cutaneous changes show great variability from pachydermia to glandular dysfunction, such as hyperhidrosis, seborrhea or acne. Pachydermia refers to the progressive thickening furrowing wrinkling of the skin on the face and forehead. In severe cases, the scalp can be involved and manifests as cutis verticis gyrate. Several patients can present with ciliary body hirsutism and blepharoptosis, mainly caused by meibomian gland hypertrophy. Hyperhidrosis usually appears in the hands and is sometimes accompanied by palmoplantar keratoderma. Regarding skeletal manifestations, periostosis often affects long bones such as the radial, ulnar, tibial, metacarpal or metatarsal bones. Symmetric and widely distributed osseous involvement is a typical finding in PHO (70). Radiographically, periostosis can be assessed in three aspects: the number of affected bones, the sites of involvement and the type of new bone formation. Acro-osteolysis can sometimes be seen on the radiographs of the extremities. The joints in PHO patients are often affected, and articular manifestations include joint swelling and stiffness, sometimes concomitant with arthritis. The knee joint is most often involved, followed by the ankle and wrist. In addition, gastrointestinal adenomas can be seen in several cases. Common gastrointestinal abnormities include diarrhea, chronic gastritis, peptic ulcer or bleeding. Anemia, which might be caused by gastrointestinal hemorrhage or myelofibrosis, is the major complication of PHO (32). Other mechanisms, such as hypoerythropoiesis, may explain the development of anemia in patients without gastrointestinal hemorrhage or myelofibrosis. However, the mechanism remains unknown. Moreover, other rare clinical manifestations, such as hypokalemia, have been reported in several cases (71). The biological measurement of PHO patients showed increased bone-formation marker levels, such as total alkaline phosphatase, bone alkaline phosphatase, the amino terminal propeptide of type I procollagen, and osteocalcin. The most important biological indicator of PHO is the PGE2 and PGE-M levels in the urine. The urinary PGE2 levels were elevated regardless of subtype. It was notably higher in PHOAR2 patients than in PHOAR1 and PHOAD patients. The urinary levels of PGE-M can also help us distinguish PHO subtypes, as it usually decreases in *HPGD*-deficient patients and increases in *SLCO2A1*-deficient patients. The urinary PGE2/PGE-M (E/M) ratio in *HPGD*-deficient patients is higher than that in normal people, but in *SLCO2A1*-deficient patients, it is similar to that in normal people. Serum PGE2 levels are mildly elevated in PHO patients, serum PGE-M levels are decreased in PHOAR1 patients and increased in PHOAR2 patients (72). Thus, these two biomarkers can also be used for diagnosis and differential diagnosis for PHO.

### 3.1 Comparison of PHO subtypes

PHO subtypes can be differentiated from each other by clinical and biological features. First, the age of onset differs in PHO subtypes, which was described as two peaks: the postnatal period and at puberty. PHOAR1 patients usually develop this disease after birth, with the median onset age of 2 year old according to our study (73), while the onset of disease symptoms in PHOAR2 and PHOAD patients is mostly in their adolescents. Second, there exists a sexual difference among subtypes. In PHOAR1 patients, the male to female ratio is approximately 1:1; however, PHOAR2 and PHOAD patients are almost all males. Only a few female PHOAR2 individuals have been reported (74), and they do not have skin or skeletal involvement. Several cases have reported female individuals with *SLCO2A1* gene mutations who present with PHO symptoms postmenopausal. This indicates that sex hormones may play a role in the development of PHO. In addition, for clinical manifestation differences between subtypes, pachydermia and clubbing fingers are usually milder in PHOAR1 and PHOAD patients than in PHOAR2 patients. PHOAR2 patients are more likely to develop gastrointestinal symptoms than PHOAR1 individuals. In addition, a higher frequency of patent ductus arteriosus (PDA) and cranial suture defects are present in PHOAR1 patients, while PHOAR2 individuals have a higher frequency of severe anemia due to myelofibrosis (75). The differences between subtypes have been listed in **Table 1**.

**Table 1:** Comparison of PHO subtypes.

		PHOAR1	PHOAR2	PHOAD
Median onset age		In childhood	In puberty	In puberty
Sex ratio (male: female)		Approximately 1:1	Almost all males	Almost all males
Bone, skin and joint symptoms		Mild	More severe than other two subtypes	Mild
Symptoms besides bone, skin and joint	Clubbing fingers	Occurs in almost all patients and the severity is independent of genotype		
	Gastrointestinal manifestations	Usually presents as diarrhea, bleeding or ulcers are rare	Bleeding and ulcers are more common and usually more severe than the other two types	Usually presents as diarrhea, bleeding or ulcers are rare
	Anemia	Less frequent	More common than the other two types	Less frequent
	Patent ductus arteriosus	Rare in all subtypes		
Urinary PGE2		↑	↑↑	↑
Urinary PGE-M		↓	↑↑	↑

↑: Above the normal range; ↑↑: Above the normal range and higher than the other two subtypes; ↓: Lower than the normal range.

### 3.2 Chronic enteropathy associated with *SLCO2A1* gene

When PHO patients have manifestations such as anemia, abdominal pain, and hypoproteinemia, clinicians should consider another hereditary enteropathy: chronic enteropathy associated with the *SLCO2A1* gene (CEAS), which shares the same causal gene with PHOAR2 (76). CEAS patients develop multiple small intestinal nonspecific ulcers, and the symptoms include abdominal pain, diarrhea, anemia, hypoproteinemia and gastrointestinal bleeding. The most affected site is the ileum, followed by the jejunum and duodenum. Contrary to PHO, it has a female predisposition. Both CEAS and PHOAR2 patients with *SLCO2A1* deficiency have elevated levels of urinary PGE2. It is suggested that the multiple small intestinal ulcers may be caused by elevated PGE2 levels in the microenvironment. However, PHOAR1 patients are absent from intestinal involvement since the PGE2 levels were also elevated. Animal experiments demonstrated that *SLCO2A1* deficiency increased PGE2 concentrations in colon tissue, thus resulting in exacerbated colitis. A study showed that *SLCO2A1* deficiency led to a high concentration of the PGE2 metabolic pathway, resulting in exacerbated intestinal inflammation *via* inflammasome activation in macrophages, which might be a possible mechanism (77). Recently, a study showed that stromal cell generated PGE2 suppresses immune cell activation during colitis propagation (78). Further investigations will be needed to clarify the underlying mechanism of CEAS.

## 4 Management

Although PHO is a rare disease, clinicians can still encounter patients with a suspected diagnosis of PHO in clinical practice since clubbing is usually associated with a variety of disorders. Because of the involvement of the joint and skin, PHO patients always go to the rheumatology department, orthopedics department or dermatology department for help. Thus, it is necessary for clinicians to be familiar with this disease and pay attention to patients with such clinical characteristics to make the right diagnosis. Under this circumstance, unfamiliar with this clinical entity may lead to misdiagnosis, thus affecting diagnosis and treatment. In this review, we hope to provide clinicians with best-practice management so that they can treat patients with PHO symptoms better.

### 4.1 Diagnosis and differential diagnosis

Clubbing is the most typical physical sign that is present in almost all patients. This finding reminds clinicians to consider the possibility of hypertrophic osteoarthropathy. With pachydermia, these two changes in appearance, which need no extra examinations, provide clinicians with clues for HOA. Imagological and biochemical examinations should be performed after physical examination. The diagnosis of hypertrophic osteoarthropathy can be preliminarily determined in patients presenting with such manifestations. Although the unique clinical signs make this disease easy to recognize, there exist several disease entities that should be distinguished from PHO. When evaluating the appearance of PHO patients, other diseases that can cause finger enlargement, such as acromegaly or pseudo-clubbing caused by secondary hyperparathyroidism, scleroderma or sarcoidosis, should be distinguished. Other skeletal or skin symptoms in PHO may also overlap with those seen in acromegaly.

The secondary form should be taken into consideration first since it is more common than the primary form. Clinicians should check if there exists a primary disease, especially in the pulmonary system. The secondary form shares similar features with PHO. It occurs as a secondary manifestation of chronic pulmonary or cardiovascular disease, more commonly as a form of paraneoplastic syndrome associated with different types of tumors, particularly lung cancer. In addition, extrathoracic conditions such as gastrointestinal tumors and infections, cirrhosis, and inflammatory bowel disease are also causes of SHO. Therefore, clinicians should pay attention to the diagnosis of SHO because it may indicate certain malignancies that require early intervention and treatment.

### 4.2 Treatment

Nonsteroid anti-inflammatory drugs (NSAIDs) can inhibit COX and result in reduction of prostaglandins, making them a proper choice for the treatment of PHO. The efficacy and safety of NSAIDs in the treatment of PHO has been confirmed by several clinical studies (72, 79). We have done a clinical intervention using etoricoxib, a selected COX-2 inhibitor, for 6 months in 41 PHO patients. After the treatment, the patients’ urinary PGE2 levels significantly decreased. And the patients showed remission of symptoms, mostly clubbing fingers, pachydermia and arthritic symptoms, but periostosis did not seem to be relieved after treatment (80) (**Table 2**). NSAIDs have limitations and side effects, especially gastrointestinal reactions such as stomach pain, making it impossible for some patients to tolerate long-term use. In several HOA cases, bisphosphonates have been used and showed effectiveness in alleviating symptoms (81). The treatment of anemia in PHO patients can always be a challenge for clinicians since NSAIDs have shown no effectiveness. The most important thing in treating anemia is to determine the cause and give corresponding therapy. For patients with unknown causes, iron therapy showed effectiveness. Severe cases require blood transfusion. In addition, evidence shows that steroid therapy can also be one choice (82). Several studies reported that botulinum toxin and plastic surgery were used for treating pachydermia (83), but according to our clinical observation, patients who accepted plastic surgery usually show facial stiffness present with a “mask face”. COX-2 inhibitor can also be used to treat SHO since it may share the same mechanism with the primary form. We have used etoricoxib for treating several SHO patients in our clinical practices. And the symptoms, especially clubbing fingers, relieved after treatment, which indicates the effectiveness of COX-2 inhibitors in treating SHO patients.

**Table 2:** Response of PHO symptoms to COX-2 inhibitor.

Symptoms	Response to COX-2 inhibitor
Digital clubbing	Notable improvement
Pachydermia	Notable improvement
Periostosis	No relief
Joint swelling and arthralgia	Notable improvement
Anemia	No relief
Gastrointestinal bleeding and ulcer	No relief and even worse

## 5 Animal models for PHO

Unfortunately, there are currently no stable animal models for PHO. Naturally, *SLCO2A1* ^-/-^ mice cannot survive because of artery ductus disclosure. Although it can be rescued by indomethacin, no skeletal phenotypes of PHO in *SLCO2A1* ^-/-^ mice have been reported to date (13). Mice with conditional knockout of *SLCO2A1* gene in macrophages were more susceptible to DSS-induced colitis (77), which is related to the intestinal phenotype of PHO. Likewise, *HPGD* ^-/-^ mice also die shortly after birth because of defective remodeling of the ductus arteriosus [24]. In addition to genetic strategies, small molecular compounds that can inhibit PGT or 15-PGDH may also be used to generate PHO models. For example, a 15-PGDH inhibitor, sw033291, was reported to have the ability to promote muscle strength and tissue regeneration. For skeletal phenotypes, mice injected with sw033291 show increased bone volume (68). In addition, direct injection of PGE2 can cause both bone loss and bone gain, depending on the method of administration (67). Although these methods show great impact on bone, none of them mimic the unique bone phenotype of PHO. We are still looking for suitable animal models accessible for systematic study of this disease, so we can learn the mechanism of this disease better and find more effective drugs with fewer side effects.

## 6 Probable therapeutic options in the future

We are still looking for better therapeutic options for this disease since COX-2 inhibitors show several side effects, such as gastrointestinal reactions. Since the PGE2 synthesis process has several rate-limiting enzymes, drugs that target enzymes other than COX-2 may be an option. PGES is the terminal enzyme that catalyzes PGH2 to PGE2. Studies have been performed to find proper candidate compounds to inhibit PGES, thus reducing PGE2 synthesis (84), which may be used to treat PHO in the future. In addition to reducing prostaglandin synthesis, the effects of elevated prostaglandin levels can be reduced by blocking the action of prostaglandins. The EP4 receptor is the main receptor of prostaglandins on bone, as COX-2 inhibitors showed nearly no relief from periostosis in PHO patients. We wondered if EP4 receptor antagonists, which have been used in treating cancer (85), can be used as an option for treating bone phenotypes in PHO patients. In addition, there remain several PHO patients who present with typical symptoms without detection of known causal genes. The whole-genomic sequencing may help us find other genes that are responsible for this disease, which may lead to novel mechanisms for the development of this disease and novel treatment. More research on animal or cell models should be performed to determine this.

## Author contributions

QL, YX and ZeZ revised the literature and draft the manuscript. SL and ZhZ revised the manuscript. All authors contributed to the article and approved the submitted version.
